# Pre-CBT resting-state connectivity and white matter integrity in OCD remission: A multimodal MRI study

**DOI:** 10.1016/j.ynirp.2025.100275

**Published:** 2025-07-08

**Authors:** Yuki Ikemizu, Yuko Isobe, Yusuke Sudo, Junko Ota, Ritu Bhusal Chhatkuli, Tubasa Sasaki, Kohei Kurita, Tokiko Yoshida, Koji Matsumoto, Masaru Kuno, Naoko Kato, Akiko Nakagawa, Eiji Shimizu, Yoshiyuki Hirano

**Affiliations:** a Research Center for Child Mental Development, Chiba University, Chiba, Japan; b Department of Cognitive Behavioral Physiology, Graduate School of Medicine, Chiba University, Chiba, Japan; c United Graduate School of Child Development, Osaka University, Suita, Japan; d National Institutes for Quantum Science and Technology, Chiba, Japan; e Department of Radiology, Chiba University Hospital, Chiba, Japan

**Keywords:** Obsessive-compulsive disorder, Cognitive-behavioral therapy, Resting-state fMRI, fc-MVPA, Diffusion MRI, Tractography, Prediction

## Abstract

**Background:**

Obsessive-compulsive disorder (OCD) is commonly treated with cognitive-behavioral therapy (CBT), yet many patients fail to achieve remission. Neuroimaging markers, such as pre-treatment functional and structural connectivity, may help elucidate OCD pathology and CBT mechanisms, and predict treatment outcomes. This study investigates the relationship between pre-treatment functional and structural connectivity and remission status in OCD patients following CBT.

**Methods:**

Thirty-three OCD patients underwent multimodal MRI, including resting-state fMRI to assess pre-treatment functional connectivity and diffusion tensor imaging (DTI) to evaluate white matter integrity. Functional connectivity multivariate pattern analysis (fc-MVPA) identified patterns linked to treatment outcomes. TRACULA, a probabilistic tractography technique, analyzed white matter tracts, focusing on diffusion metrics such as fractional anisotropy (FA), mean diffusivity (MD), radial diffusivity (RD), and axial diffusivity (AD). Analysis of covariance (ANCOVA) was used to examine group differences.

**Results:**

Remission was associated with significantly higher pre-treatment resting-state functional connectivity between the occipital pole and lateral occipital cortex (height threshold: p < 0.001 uncorrected and cluster threshold: p < 0.05 cluster-size FDR corrected for multiple comparisons), suggesting a role in visual processing. Differences in white matter integrity were found in the corpus callosum rostrum, left acoustic radiation, right dorsal cingulum bundle, and right superior longitudinal fasciculus II, though these results were not corrected for multiple comparisons.

**Conclusion:**

Enhanced pre-treatment functional connectivity in visual processing regions and specific white matter tracts may serve as biomarkers for remission in OCD following CBT. These findings could improve understanding of CBT’s neural effects and guide personalized treatment strategies.

## Highlights

• rsFC extracted from resting-state fMRI evaluates brain functional connectivity.
• Fc-MVPA provides an unbiased approach to analyzing specific rsFC patterns.
• Diffusion tensor imaging (DTI) assesses white matter integrity.
• Integration of rsFC and white matter revealed involvement in visual processing.
• We identified potential predictors of OCD remission following CBT treatment.

## Graphical abstract

**Figure:**
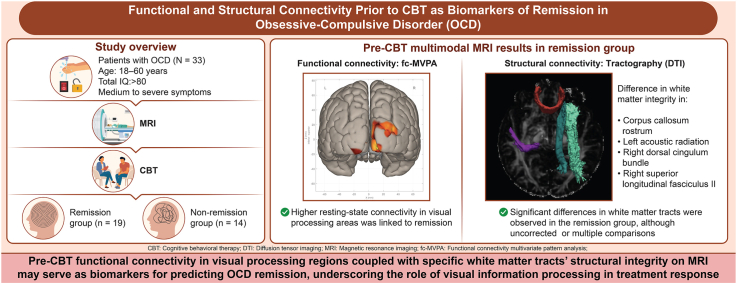

## 1 Introduction

Obsessive-compulsive disorder (OCD) is a common, chronic, and often disabling condition classified as an obsessive-compulsive and related disorder and characterized by obsessions and compulsions (Diagnostic and Statistical Manual of Mental Disorders, Fifth Edition, Text Revision*,* DSM-5-TR, American Psychiatric Association, 2022). Obsessions are unwanted, intrusive thoughts, images, impulses, or urges that persist and recur, and are typically associated with anxiety (Stein et al., 2019). Compulsions include behaviors such as handwashing, repeated checking, strict organization, counting, praying, or repeating words or phrases (DSM-5-TR). These repetitive actions follow rigid rules and are performed in response to obsessions or to achieve a sense of “completeness” (Stein et al., 2019).

Cognitive-behavioral therapy (CBT) is the first-line treatment for OCD (Stein et al., 2019), including exposure response prevention (ERP), as recommended by the National Institute for Health and Care Excellence (NICE) guidelines. ERP involves psychoeducation, exposure to feared thoughts, and resistance to compulsive behaviors (Law and Boisseau, 2019). For example, one meta-analysis demonstrated significant symptom reduction with CBT with ERP (Reid et al., 2021). In a meta-analysis of CBT for adult patients with OCD, remission was typically defined using a cut-off score of ≤12 on the Yale-Brown Obsessive Compulsive Scale (Y-BOCS; Goodman et al., 1989), with a reported remission rate of only 59.2 % (Öst et al., 2022). This relatively low remission rate may reflect the complex nature of OCD and evolving understanding of the mechanisms underlying CBT.

Consequently, neuroimaging studies using magnetic resonance imaging (MRI) have garnered significant attention because of their potential to clarify the neural mechanisms underlying OCD. Among these, two of the most commonly used modalities are resting-state functional MRI (rsfMRI), which evaluates functional connectivity (FC), and diffusion tensor imaging (DTI), which estimates structural connectivity through white matter tracts. The cortico-striato-thalamo-cortical (CSTC) circuit is a well-established focus in OCD research, known for its structural and functional abnormalities (Bruin et al., 2023; Milad and Rauch, 2012). Five primary circuits have been implicated in OCD: the sensorimotor, dorsal cognitive, ventral cognitive, ventral affective, and fronto-limbic circuits (van den Heuvel et al., 2016). However, alterations in networks beyond the CSTC circuit, such as the fronto-limbic, frontoparietal, and cerebellar networks, have also been observed (Bruin et al., 2023; Lillevik Thorsen et al., 2020; Sha et al., 2020; Stern et al., 2012). Thus, whole-brain studies, rather than research focusing solely on the CSTC circuit, are increasingly needed to achieve a comprehensive understanding of OCD pathology.

In this context, resting-state FC (rsFC), derived from rsfMRI, assesses the statistical dependencies between fluctuations in blood oxygenation level-dependent (BOLD) signals across anatomically distinct brain regions or networks (Biswal et al., 1995). In OCD, reduced rsFC in the default mode network (DMN) has been associated with greater symptom severity, whereas increased rsFC in the frontoparietal network correlates with clinical scores, and lateral prefrontal rsFC relates to specific symptom dimensions (Fornaro and Vallesi, 2023). The OCD working group of the Enhancing Neuro-Imaging Genetics through Meta-Analysis (ENIGMA), known for using a large global sample, conducted a comprehensive mega-analysis of rsfMRI data (Bruin et al., 2023). Their findings indicate that OCD is characterized by widespread FC abnormalities, predominantly displaying global hypoconnectivity, especially within the sensorimotor network, with limited regions of hyperconnectivity involving the thalamus (Bruin et al., 2023).

Multivariate pattern analysis (MVPA) has recently been introduced to mitigate the potential risk of false negatives inherent in hypothesis-driven approaches, such as selecting specific regions of interest (ROIs) or seed regions, by enabling whole-brain data-driven analyses without predefined assumptions (Nieto-Castanon, 2022). FC multivariate pattern analysis (fc-MVPA) builds on this approach by extending MVPA methods to infer brain-wide connectome patterns (Nieto-Castanon, 2022). For example, a previous study conducted with 38 participants with social anxiety disorder reported that rsFC in the amygdala combined with initial Liebowitz Social Anxiety Scale scores accounted for 33 % of the variance in CBT treatment outcomes, which increased to 50 % when including MVPA results, demonstrating its enhanced predictive power (Whitfield-Gabrieli et al., 2016). Fc-MVPA has high sensitivity, as demonstrated by its ability to detect meaningful effects across the entire human connectome (Nieto-Castanon, 2022). This analytical strength enables the identification of subtle changes in FC, such as those observed in the treatment response to cognitive therapy for social anxiety disorder (Kurita et al., 2023), and in longitudinal rsFC following a single bout of acute exercise in youth (Cline et al., 2024).

In parallel, DTI offers complementary insights into structural connectivity by capturing the diffusion of water molecules along white matter fibers. Diffusion MRI (dMRI) provides a method for mapping and visualizing nerve fiber tracts by assessing the diffusive motion of protons and other metabolites within the voxel of an anisotropic medium (Basser et al., 1994). DTI, a specific type of dMRI, detects changes in radiofrequency signals as water moves toward or away from the source. In an unrestricted environment, water diffuses isotropically; however, in white matter, diffusion is primarily aligned with the direction of nerve fibers because of the constraints imposed by axonal membranes and myelin sheaths (Haghshomar et al., 2023). Consequently, DTI effectively maps the structural connectivity of the brain by tracking water movement along neural pathways (Kang et al., 2024).

DTI provides several diffusion indices. The most frequently reported measure is fractional anisotropy (FA), which is elevated in tissues with well-organized fiber structures and lower in less organized regions (Mori and Zhang, 2006). FA can be further divided into components reflecting diffusion parallel (axial diffusivity, AD) and perpendicular (radial diffusivity, RD) to the white matter tracts (Rosso et al., 2014). Mean diffusivity (MD) represents the average diffusion across all directions within the tissue and is associated with white matter development and maturity (Beaulieu, 2002).

Several studies have used DTI to investigate white matter integrity in OCD, highlighting notable findings from previous research. Clinically, an earlier age of OCD onset has been linked to reduced FA in the right thalamus and increased RD in the right corpus callosum (Rosso et al., 2014). In adult patients with OCD, significant FA reductions have been reported in the sagittal stratum and posterior thalamic radiation, with lower FA in the sagittal stratum correlated with a younger age of onset, longer illness duration, and a higher percentage of medicated patients, as noted by the ENIGMA OCD working group (Piras et al., 2021). Additionally, measures of diffusivity in the corpus callosum, internal capsule, and posterior thalamic radiation have helped distinguish between patients with OCD and healthy controls (B.-G. Kim et al., 2024).

These diffusion indices provide the basis for various analytical approaches to assess white matter integrity. Two common approaches for analyzing white matter integrity are Tract-Based Spatial Statistics (TBSS) and TRActs Constrained by UnderLying Anatomy (TRACULA), a tractography method (Kang et al., 2024). TBSS, a voxel-based technique, enables whole-brain analysis focused on a core white-matter skeleton (Olson et al., 2017; Smith et al., 2006). In contrast, diffusion tensor tractography maps white matter pathways and complements the more localized TBSS approach (Roine et al., 2015), as it is not restricted to regions with the highest FA values (Olson et al., 2017). TRACULA is a probabilistic tractography method that incorporates anatomical neighborhood regions for global analysis (Yendiki et al., 2011). This technique enables highly accurate tract reconstruction and shows promise in detecting traumatic axonal injury in individual patients (Maffei et al., 2023), offering a more refined analysis of the neurological impact of CBT.

Although informative, single-modality approaches provide a limited understanding of the neural mechanisms involved. Recently, multimodal imaging approaches that integrate morphological data, white matter integrity, and network function measurements using rsfMRI have demonstrated promise in better predicting individual treatment responses (Heuvel et al., 2022). For example, combining rsFC with structural connectivity has been effective in identifying individual differences in suicide risk among older adult patients with depression (M. Gao et al., 2023). Such methods are expected to provide a more nuanced understanding of the underlying pathophysiology (Maximo et al., 2021).

Despite this promise, relatively few studies have used multimodal imaging to investigate CBT-related treatment outcomes in OCD. Several neuroimaging studies have investigated the effects of CBT in patients with OCD. Furthermore, notable rsfMRI (Li et al., 2018; Moody et al., 2017; Rangaprakash et al., 2021; Reggente et al., 2018; Yang et al., 2015) and DTI (Cao et al., 2021; Zhong et al., 2019) studies have been conducted. However, these studies have primarily relied on unimodal neuroimaging analyses, potentially limiting their ability to capture the complex and multifaceted nature of treatment-related brain changes.

The primary aim of this study is to examine the relationship between pre-CBT functional and structural connectivity and remission in patients with OCD. We hypothesize that MVPA and TRACULA will show improved sensitivity compared to the methods used in previous research. This approach aims to enhance our understanding of the responsiveness of OCD to CBT by integrating both functional and structural connectivity data, potentially informing new treatment strategies.

## 2 Methods

### 2.1 Participants

**Fig. 1:**
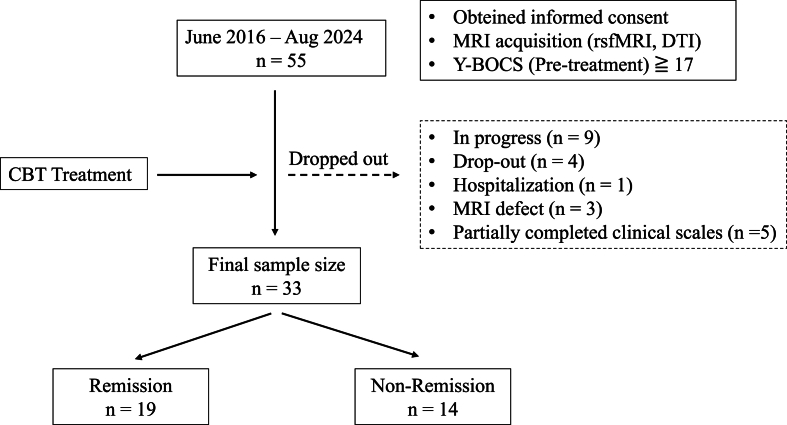
Recruiting process of this study.

We recruited patients from consecutive referrals to the OCD outpatient clinic at Chiba University Hospital, Japan, between June 2016 and August 2024. A trained psychologist diagnosed all patients with OCD based on the the Diagnostic and Statistical Manual of Mental Disorders, Fifth Edition (DSM-5) (American Psychiatric Association, 2013) or DSM-5-TR criteria. Fig. 1 provides an overview of participant enrollment and the study workflow.

Inclusion criteria required patients to be aged 18–60 years, have a total intelligence quotient (IQ) above 80 (or be assessed as not having a clinical intellectual disability), and display medium-to-severe obsessive-compulsive symptoms. IQ was evaluated using the Wechsler Adult Intelligence Scale-Revised or Japanese Adult Reading Test (Matsuoka et al., 2006). Obsessive-compulsive symptom severity was assessed using the Y-BOCS (Goodman et al., 1989; Hamagaki et al., 1999), with a total score of 16 or higher indicating medium-to-severe symptoms.

Exclusion criteria included age below 16 or above 60, presence of neurological disorders, schizophrenia, substance dependence, organic brain diseases, pregnancy, or severe physical illnesses such as cardiovascular disease or hepatitis. Among the participants, 27 (81 %) were on medication and 6 (19 %) were not. Additionally, 19 (57 %) patients had comorbid conditions, whereas 14 (43 %) did not. Detailed clinical and demographic data are presented in Table 1, and specific medications are listed in Supplementary Table 1.

**Table 1:** Demographic and clinical data of Remission and Non-Remission groups.

	Mean ± SD			
	Remission (N = 19)	Non-Remisson (N = 14)	t value	p value
Age(years)	30.16 ± 8.36	37.21 ± 9.04	2.31	0.03
Sex(male/female)	11 (57 %)	12 (86 %)		0.08^a^
Handedness(right/left/ambidextrous)	16/1/1	14/0/0		
Age of onset (years)	19.32 ± 9.50	24.93 ± 11.96	1.50	0.14
Number of CBT sessions	20.47 ± 9.20	19.36 ± 7.00	−0.108	0.92
Baseline Y-BOCS	23.6 ± 4.67	27.00 ± 5.67	1.87	0.07
obsession	12.31 ± 2.26	13.79 ± 3.07	1.59	0.12
complusive	11.32 ± 2.89	13.21 ± 2.83	1.88	0.07
aggression/checking (yes/no)	16/3	11/3		0.67^a^
clean/contamination (yes/no)	14/5	11/3		0.74^a^
sexual/religion (yes/no)	6/13	3/11		0.52^a^
hoarding (yes/no)	3/16	2/12		0.91^a^
ordening, symmetry (yes/no)	3/16	6/8		0.08^a^
Change in Y-BOCS	15.63 ± 4.42	6.29 ± 5.44	−5.44	<0.001
IQ	102.69 ± 8.81 (N = 16)	103.29 ± 10.15 (N = 14)	0.17	0.86
PHQ-9	8.56 ± 5.99 (N = 18)	11.53 ± 6.67 (N = 13)	1.31	0.20
GAD-7	8.61 ± 4.96 (N = 18)	12.77 ± 4.15 (N = 13)	2.46	0.02
Comorbidities				
ASD	5	8		0.07^a^
Bipolar Disorder or MDD	2	4		0.18^a^
GAD or Panic Disorder	0	2		0.09^a^
Medication (with/without)	14/5	13/1		0.15^a^

a Chi-square test.

Participants also completed the Beck Depression Inventory Second Edition (Beck et al., 1996; Kojima et al., 2002) and Patient Health Questionnaire-9 (Kroenke et al., 2001; Muramatsu et al., 2018). Handedness was determined using the Edinburgh Handedness Inventory (Oldfield, 1971).

### 2.2 Treatment phase

We implemented CBT for OCD using the *Manual of Cognitive Behavioral Therapy for Obsessive-Compulsive Disorder* (Nakatani et al., 2016), a treatment manual for adult outpatients. This manual was developed following earlier studies that demonstrated the efficacy of behavioral therapy for Japanese patients with OCD (Nakatani et al., 2005) and is widely used in clinical practice in Japan as the standard treatment guidelines for OCD, recognized by the Ministry of Health, Labour and Welfare. CBT sessions were scheduled for 50 min weekly. Regarding the structure of the CBT program, the first session focused on obtaining a detailed medical history. Sessions two through four provided psychoeducation about OCD and case formulation. Subsequent sessions were tailored to each patient, incorporating techniques such as exposure and response prevention (when applicable), shaping methods, and addressing the patient’s situation, including family dynamics, work conditions, and school adjustments. The final two sessions introduced relapse prevention strategies (Hamatani et al., 2020; Nakatani et al., 2016).

Although standard treatment typically consists of 16–20 sessions, adjustments were made based on each patient’s condition and progress. All therapists involved in this study completed the Chiba Improving Access to Psychological Therapies project training program (Kobori et al., 2014). The quality of CBT was ensured through weekly individual or group supervision by a psychiatrist (Hamatani et al., 2020).

### 2.3 Definition of remission

We defined clinical remission as a total Y-BOCS score below 13 at the final treatment session (Mataix-Cols et al., 2016). A decrease of 35 % or more in a patient’s Y-BOCS score indicated clinical response (Brecke et al., 2021; Mataix-Cols et al., 2016).

### 2.4 Neuroimaging acquisition

We obtained all brain images using a 3.0 T magnetic resonance scanner (Discovery MR750, GE Healthcare, Waukesha, WI, USA) equipped with a 32-channel phased-array head coil. For structural imaging, we collected a 3D magnetization-prepared rapid-acquisition gradient echo (MPRAGE) T1-weighted sequence covering the entire brain with the following parameters: repetition time (TR) of 8.128 or 8.168 ms, echo time (TE) of 3.16 or 3.18 ms, flip angle (FA) of 15° or 11°, inversion time (TI) of 420 or 400 ms, acquisition matrix of 256 × 256, slice thickness of 1.0 or 1.2 mm, and voxel size of 1 × 1 × 1 mm^3^ or 1.02 × 1.02 × 1.2 mm^3^. A gradient echo-planar imaging sequence was used for resting-state functional BOLD images: repetition time (TR = 2300 or 2500 ms), echo time (TE = 30 ms), flip angle (FA = 81° or 80°), slice thickness (3.2–3.5 mm), acquisition matrix (64 × 64), and voxel size (3.3 × 3.3 × 3.5 or 3.3125 × 3.3125 × 3.2 mm^3^). During scanning, participants were instructed to maintain focus on crosshairs projected onto the screen. Datasets with different parameters were treated as distinct data types.

### 2.5 Functional MRI data

**Fig. 2:**
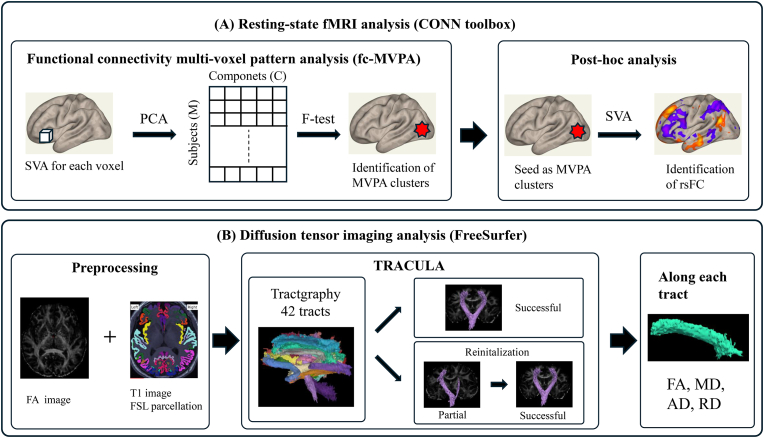
Workflow of imaging analysis. (A) Resting-state fMRI Analysis (CONN-toolbox) 1. Fucntional Connetivity Multi-voxel Pattern Analysis (fc-MVPA): Seed-to-voxel analysis (SVA) was conducted for each voxel. Principal Component Analysis (PCA) and F-test were applied to refine the data and identify MVPA (Multi-Voxel Pattern Analysis) clusters. 2. Post-hoc analysis: MVPA clusters are used as seeds for further detailed investigations into functional connectivity patterns through seed-to-voxel analysis. (B) Diffusion Tensor Imaging (DTI) Analysis (FreeSurfer) 1. Preprocessing: Utilizes FA (Fractional Anisotropy) image and T1-weighted images with FSL parcellation. 2. Tractgraphy with TRACULA: Tractography was performed using the TRACULA method. Tracts categorized as **successful** were retained, while **partial** tracts underwent reinitialization for further processing. 3. Feature Extraction: Includes FA, MD (Mean Diffusivity), AD (Axial Diffusivity), and RD (Radial Diffusivity) measurements along each tract.

Fig. 2A illustrates the neuroimaging analytical workflow of rsfMRI data.

#### 2.5.1 Functional MRI data processing

The CONN functional connectivity toolbox 22a (https://web.conn-toolbox.org/) running in a MATLAB R2024a environment (The MathWorks, Inc., Natick, MA, USA, https://jp.mathworks.com/) was used to preprocess the functional and structural images. As part of preprocessing, the first seven scans were removed for data with a repetition time (TR) of 2300 ms, and the first six scans were removed for TR = 2500 ms. The default preprocessing pipeline for volume-based analyses was then applied, which involved direct normalization to the Montreal Neurological Institute (MNI) space. The pipeline also included functional realignment and unwarping, slice timing correction (acquired in interleaved bottom-up order), outlier detection, functional segmentation and direct normalization, structural segmentation, and normalization, smoothing with a 6 mm full-width at half maximum Gaussian kernel, and band-pass filtering (0.008–0.09 Hz) (J. He et al., 2024).

#### 2.5.2 Multivariate pattern analysis

A whole-brain connectome-wide group MVPA was conducted for unbiased identification of seed regions for seed-to-voxel analysis (H. E. Kim et al., 2024). MVPA is a voxel-to-voxel measure that captures the connectivity patterns between each brain voxel and the rest of the brain. FC was computed for each voxel and then reduced via principal component analysis to highlight key between-subject differences. An F-test was used to identify voxel patterns that differentiated between the remission and non-remission groups. Detailed MVPA methods are described in the Supplementary Methods.

Following the identification of significant components, clusters were mapped using the FSL Harvard-Oxford Atlas (Smith et al., 2004) for cortical and subcortical regions and the Automated Anatomical Labeling atlas (Tzourio-Mazoyer et al., 2002) for cerebellar regions. These analyses controlled for age, sex, pretreatment Y-BOCS score, and data type (i.e., differences in scanning parameters such as TR and voxel size) as nuisance covariates to account for potential variability arising from acquisition protocol differences. Clusters were extracted using a height threshold of *p* < *0.001* (uncorrected for multiple comparisons) and a cluster-size threshold of *p* < *0.05* cluster-size false discovery rate (FDR; corrected for multiple comparisons).

#### 2.5.3 Seed-to-voxel analysis

MVPA is an omnibus test; therefore, follow-up post hoc analyses were needed to further characterize connectivity differences using significant clusters identified as ROIs in seed-to-voxel analyses, consistent with previous research (e.g., (Weber-Goericke and Muehlhan, 2023; Whitfield-Gabrieli et al., 2016). Group-level analyses were conducted as post hoc tests in second-level FC analyses to assess the significance of the FC. These analyses controlled for age, sex, pretreatment Y-BOCS scores, and data type as nuisance variables. The thresholds for FC analysis were set at a height threshold of *p* < *0.001* (uncorrected for multiple comparisons) and cluster-sized threshold of *p* < *0.001* (FDR, corrected for multiple comparisons).

### 2.6 Structural MRI data processing for TRACULA

Cortical reconstruction and volumetric segmentation were performed using FreeSurfer version 7.4.1, a widely used software package for analysis and visualization (https://surfer.nmr.mgh.harvard.edu). The “recon-all” function in FreeSurfer processed all subject images. Subcortical segmentation was performed using MATLAB R2024a (Iglesias et al., 2018). Quality control included reviewing skull-stripping and automated segmentation of gray and white matter boundaries using Freeview and the “ENIGMA-FreeSurfer-protocol” shell script (https://github.com/ENIGMA-git/ENIGMA-FreeSurfer-protocol). Cortical parcellation and subcortical segmentation were verified for proper implementation following the “ENIGMA Cortical Quality Control Guide 2.0 April 2017” (https://enigma.ini.usc.edu/wp-content/uploads/2010/05/ENIGMA_Cortical_QC_2.0.pdf).

### 2.7 Diffusion MRI data

Fig. 2B depicts the analytical process for DTI.

#### 2.7.1 Diffusion MRI processing

Diffusion-weighted images were processed by removing the skull and correcting for eddy-current distortions and motion using FSL 6.0.1 (Andersson and Sotiropoulos, 2016). The diffusion tensor model was applied to the data using DTIFIT, while the ball-and-stick model was fit with BEDPOSTX (Behrens et al., 2003) in FSL. Automated reconstruction of 42 major white matter pathways was performed using the global probabilistic tractography algorithm TRACULA in FreeSurfer 7.4.1 (Maffei et al., 2021; Yendiki et al., 2011). TRACULA estimates neural pathways by integrating the “ball-and-stick” diffusion model with existing anatomical knowledge (Yendiki et al., 2011). Four diffusion metrics—FA, AD, RD, and MD—were derived for each tract. All image processing, including T1 and DTI, was performed using an Ubuntu 22.04-based Lin4Neuro system (Nemoto et al., 2011).

#### 2.7.2 Quality control for TRACULA

Alignment of the principal direction vectors with the white matter tracts was confirmed through visual inspection of the raw FA images, following the Protocol for FA and Vector Alignment QC Analysis for ENIGMA-DTI (https://github.com/ENIGMA-git/ENIGMA_DTI_03_Quality_Control). The resulting 42 reconstructed tracts were further inspected based on the quality control pipeline (X. He et al., 2021) to ensure that there were no failed or partial reconstructions. For tracts that exhibited volumes below the specified threshold or showed errors, TRACULA was rerun with reinitializing of the control points (reinit parameter set to 1), as recommended (Maffei et al., 2023; Yendiki et al., 2011). Any tracts that continued to display issues after reinitialization were excluded from subsequent analyses.

### 2.8 Statistical analysis

Differences in age and clinical scores among patients with OCD were evaluated using a two-sample *t*-test. The chi-square test was used to identify intergroup differences in sex and data type, with the significance threshold set at *p* < *0.05*. Analysis of covariance (ANCOVA) was used to examine DTI metrics (FA, MD, AD, and RD) in the target white matter tracts, controlling for age, sex, pretreatment Y-BOCS score, intracranial volume, and data type (defined by imaging parameters such as TR and voxel resolution) to control for any effects related to variations in scanning protocols. Multiple comparisons were adjusted using the Bonferroni correction, with *p* < *0.00030* considered statistically significant. All statistical tests were two-tailed, and analyses were conducted using Python 3.10 (Python Software Foundation, https://www.python.org).

### 2.9 Statement of ethics

Ethical approval for this study was obtained from the Research Ethics Committee of the Graduate School of Medicine, Chiba University (M10545) and was conducted in accordance with the principles outlined in the Helsinki Declaration. The study was carried out only after sufficient explanation and written informed consent was obtained from all participants. All methods were performed in accordance with relevant guidelines and regulations. The trial was registered as UMIN000024087.

## 3 Results

### 3.1 Demographic and clinical characteristics

The results of a chi-square test showed no significant difference in the sex ratio between groups. In the remission group, 57 % were female and 43 % were male; in the non-remission group, 86 % were female and 14 % were male (*p* = *0.08*). Although the remission group had a relatively younger age of onset, the difference was not statistically significant. The remission group had a significantly lower mean age of 30.16 years (±9.04) compared to 37.21 years (±8.34) for the non-remission group (*t* = *2.31, p* = *0.027*). The average number of CBT sessions was 19.67 (±8.74) in the remission group *(n* = 18) and 19.36 (±7.00) in the non-remission group, with no significant difference between groups (*t* = *-0.108, p* = *0.92*). Regarding clinical measures, the mean Y-BOCS score at baseline was 23.6 (±5.67) in the remission group and 27 (±4.67) in the non-remission group (*t* = *1.87, p* = *0.07*). No significant differences were observed in the obsession and compulsion sub-scores between groups. Symptom profiles, including aggression/checking, cleaning/contamination, sexual/religious obsessions, hoarding, and ordering/symmetry, were comparable, with *p*-values ranging from 0.08 to 0.91. Mean IQ scores were similar between groups: 102.69 (±8.81) in the remission group (*n* = 16) and 103.29 (±10.15) in the non-remission group (*n* = 14), with no significant difference (*t* = *0.17, p* = *0.86*).

### 3.2 Resting-state fMRI analysis

#### 3.2.1 fc-MVPA results

**Fig. 3:**
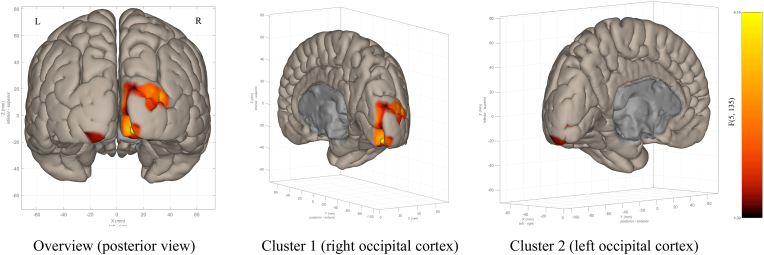
Resulting clusters from fc-MVPA analysis using Remission > Non-remission contrast. Covariate: age, sex, pre-treatment Y-BOCS, data type. Voxel threshold p < 0.001 (p-uncorrected), Cluster threshold p < 0.05 (cluster-size p-FDR corrected).

The MVPA identified a significant cluster in the bilateral occipital cortex, demonstrating distinct connectivity patterns between the remission and non-remission groups (Table 2 and Fig. 3). This cluster included the right occipital pole and occipital fusiform gyrus (peak MNI coordinates: 14, −94, −8; cluster size k = 498) and the left occipital pole and occipital fusiform gyrus (−16, −86, −6; cluster size k = 176). However, comparisons between the response group, defined as those with a decrease of 35 % or more in Y-BOCS score, and the non-response group, as well as a correlation analysis with changes in Y-BOCS scores, did not yield any significant clusters.

**Table 2:** Clusters extracted from MVPA.

Regions	x	y	z	Custer size	size p-FWE	size p-FDR	F value
Occiptial Pole Right, Occipital Fusiform Gyrus Right	14	−94	−8	498	0.000000	0.000000	5.45
Occipital Pole Left, Occipital Fusiform Gyrus Left	−16	−86	−6	176	0.000000	0.000000	6.2

#### 3.2.2 Post hoc seed-to-voxel analysis of fc-MVPA-derived clusters

**Fig. 4:**
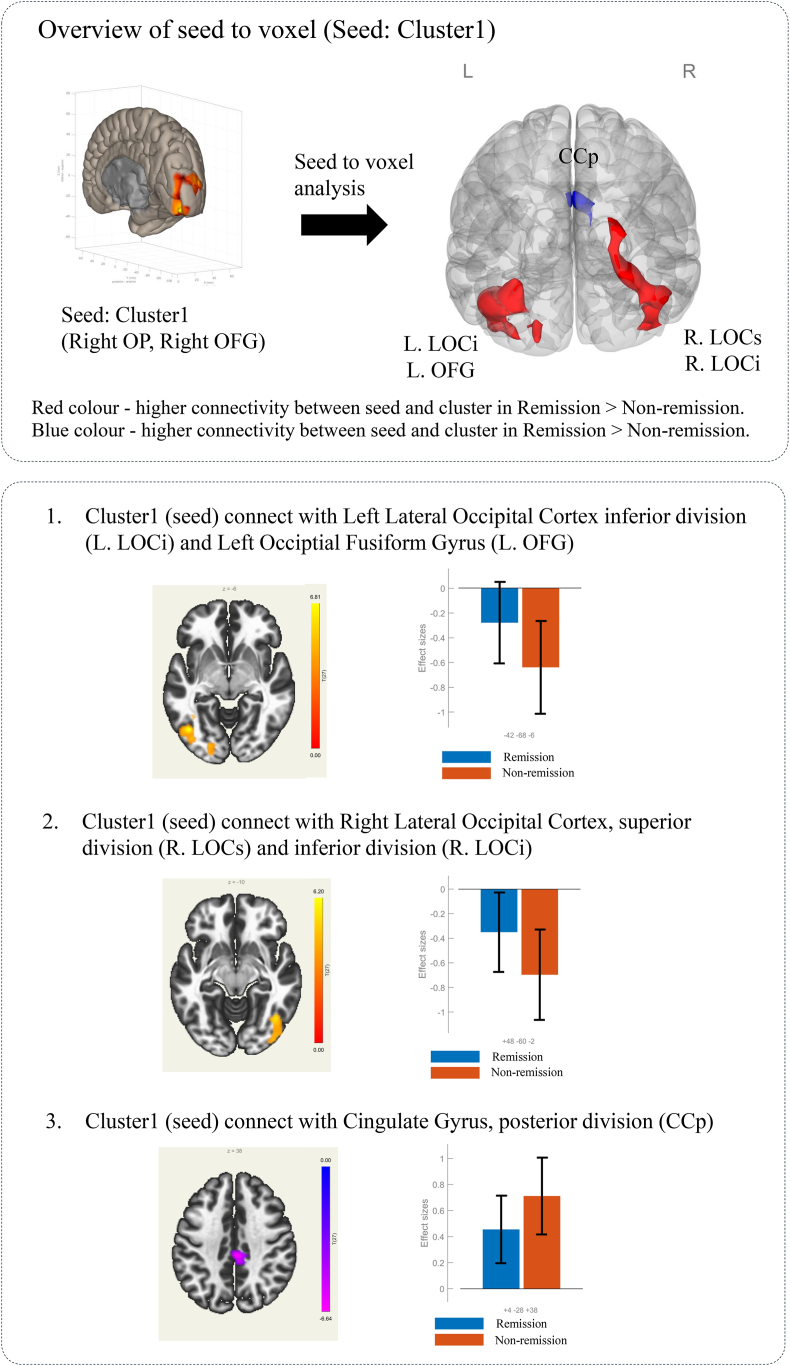
Resulting clusters from seed-based analysis using cluster1 as the seed. Covariate: age, sex, pre-treatment Y-BOCS, data type. Voxel threshold p < 0.001 (p-uncorrected), Cluster threshold p < 0.001 (cluster-size p-FDR corrected).

**Fig. 5:**
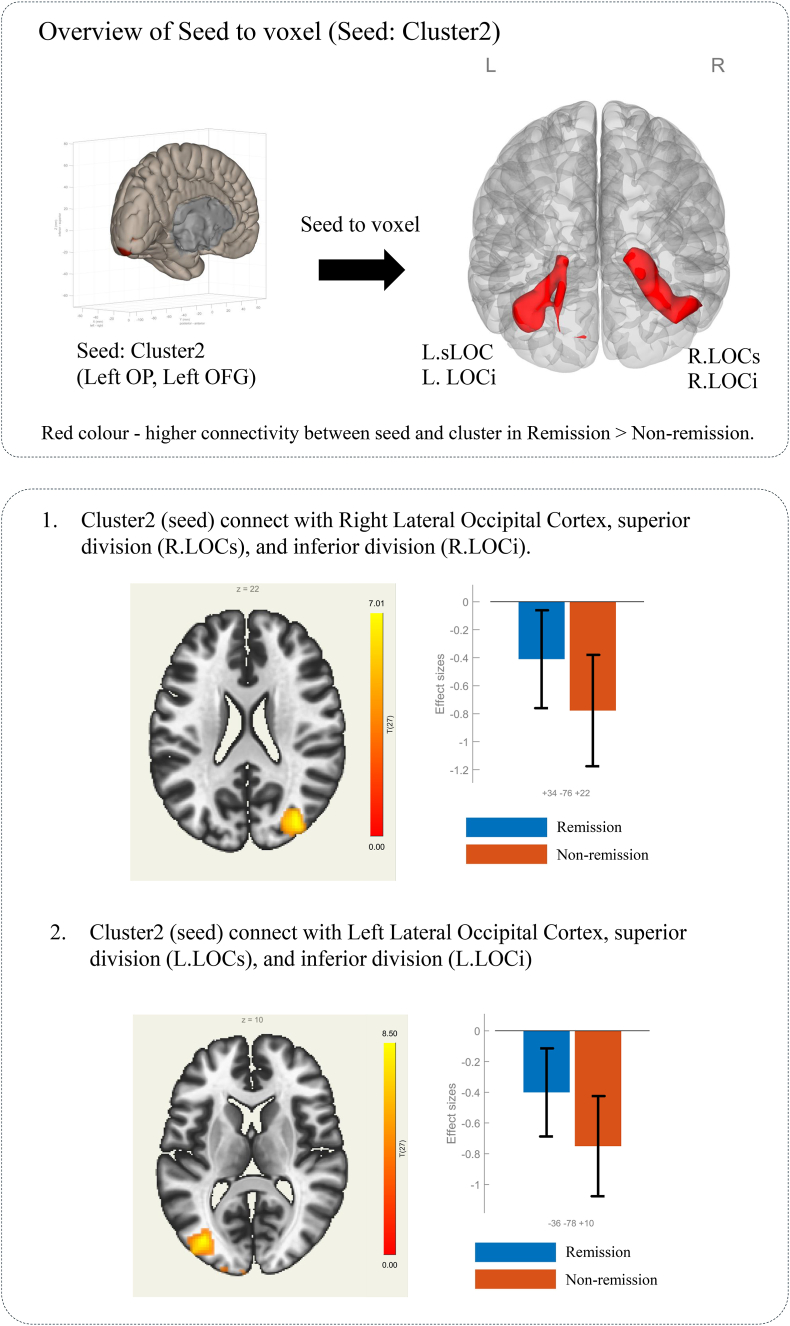
Resulting clusters from seed-based analysis using cluster 2 as the seed. Covariate: age, gender, pre-treatment Y-BOCS, data type. Voxel threshold p < 0.001 (p-uncorrected), Cluster threshold p < 0.001 (cluster-size p-FDR corrected).

As presented in Table 3, a seed-to-voxel rsFC analysis was performed using the MVPA-derived clusters as seeds. When the significant cluster in the right occipital cortex was used as a seed, patients in the remission group exhibited significantly different rsFC patterns than those in the non-remission group (Fig. 4). These differences included enhanced connectivity between the right occipital cortex (seed) and several brain regions, including the bilateral lateral occipital cortex, left fusiform gyrus, and cingulate gyrus, compared with the non-remission group. Similarly, when the left occipital cortex was used as a seed, a comparison between the remission and non-remission groups revealed significant differences in rsFC, notably between the left occipital cortex (seed) and bilateral lateral occipital cortex (Fig. 5).

**Table 3:** Seed-based analysis results using clusters extracted from fc-MVPA, Remission > Non-Remission contrast.

Seed	Regions	x	y	z	Custer size	size p-FWE	size p-FDR	T value
Cluster1(Right Occiptial Pole, Right, Occipital Fusiform Gyrus)	Lateral Occipital Cortex, inferior division, Left	−42	−68	−86	1480	0.000000	0.000000	6.89
	Occiptial Fusiform Gyrus, Left							
	Lateral Occipital Cortex, superior division Right	48	−60	−2	1394	0.000000	0.000000	6.73
	Lateral Occipital Cortex, inferior division Right							
	Cingulate Gyrus, posterior division	4	−28	38	259	0.000036	0.000025	−6.22
Cluster2(Left Occipital Pole, Left Occipital Fusiform Gyrus)	Lateral Occipital Cortex, superior division Right	34	−76	22	1457	0.000000	0.000000	6.6
	Lateral Occipital Cortex, inferior division Right							
	Lateral Occipital Cortex, superior division Left	−36	−78	10	1204	0.000000	0.000000	7.67
	Lateral Occipital Cortex, inferior division Left

### 3.3 Diffusion tensor imaging analysis

#### 3.3.1 Quality assessment and feasibility of tractography reconstructions

Following the application of TRACULA to all participants, some tracts were flagged as poorly reconstructed because their volume was less than twice their length or they failed visual inspection. A total of 3.7 % of all tracts (51 tracts, 42 tracts × 33 participants) had reconstruction issues. Certain tracts did not exhibit any reconstruction failures, whereas others had a higher failure rate. The anterior commissure exhibited the highest rate of reconstruction failure (42 % of participants), followed by the left (27 %) and right fornix (24 %). Other tracts with higher failure rates included the right corticospinal tract, right frontal aslant tract, right superior longitudinal fasciculus (SLF) I, left SLF III, and left uncinate fasciculus, which all showed a 6.1 % failure rate. Additionally, tracts such as the left anterior thalamic radiation, right anterior thalamic radiation, right arcuate fasciculus, right cingulum bundle (ventral), corpus callosum body (premotor), corpus callosum rostrum, corpus callosum splenium, left extreme capsule, left frontal aslant tract, and left SLF I had a 3.0 % failure rate. After reinitialization, 1.4 % of all tracts (20 tracts, 42 tracts × 33 participants) were still flagged as poorly reconstructed. These tracts were excluded from the analysis. The breakdown of these failed reconstructions was as follows: anterior commissure (failed in 33 %), left fornix (15 %), and right fornix (12 %).

#### 3.3.2 Group differences in white matter integrity using TRACULA

**Fig. 6:**
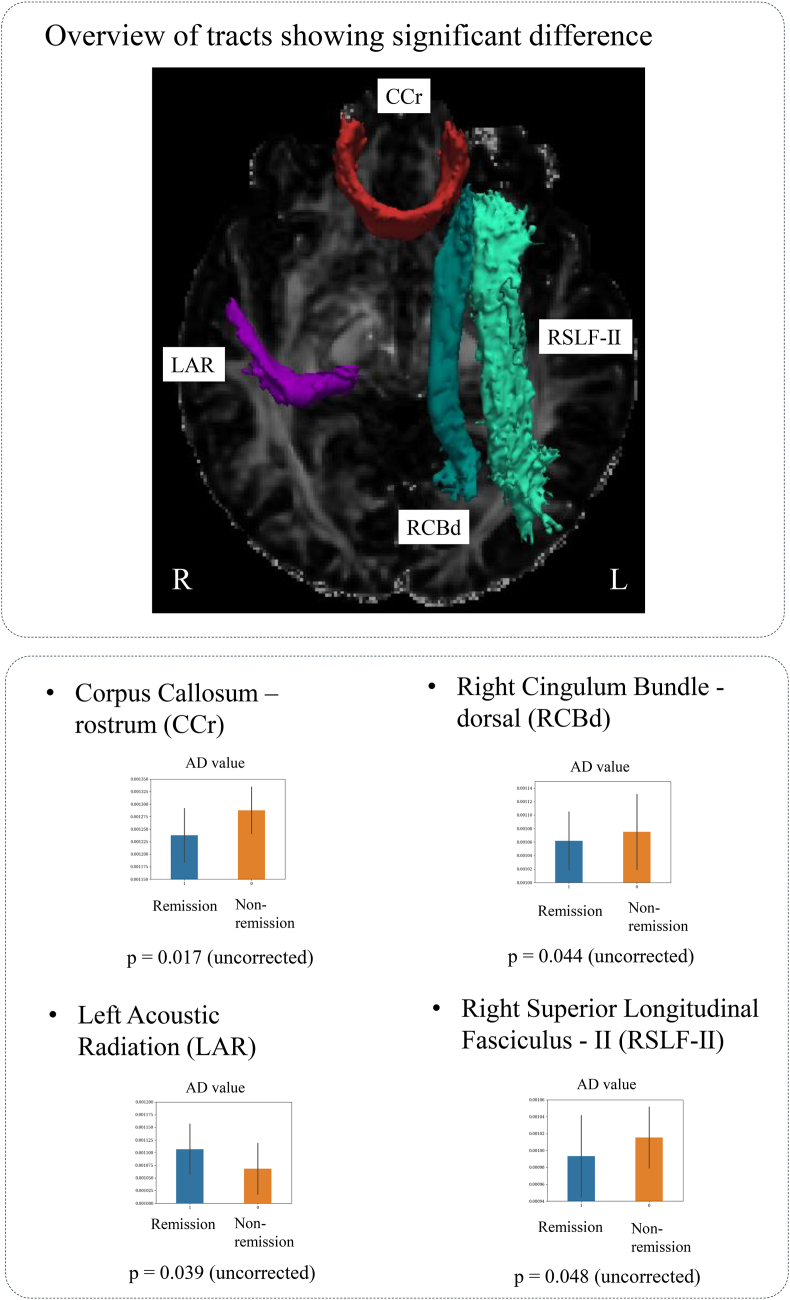
Diffusion indices of white matter tracts which showed significant difference between the remission and non-remission groups. This figure displays diffusion indices of white matter tracts that demonstrated significant differences between the remission and non-remission groups. The analysis was conducted using analysis of covariance (ANCOVA), controlling for age, sex, pre-treatment Y-BOCS, intracranial volume (ICV), and data type.

We performed an ANCOVA between the remission and non-remission groups, focusing on key diffusion metrics, such as FA, MD, RD, and AD. Bonferroni-corrected multiple comparisons revealed no significant differences (*p* < *0.00030*). However, significant differences in the AD (average center) values were observed for the following four tracts (*p* < *0.05*): corpus callosum rostrum, left acoustic radiation, right cingulum bundle dorsal, and right SLF II (Table 4 and Fig. 6).

**Table 4:** Tract diffusion imaging metrics between Remission and Non-remission groups ANCOVA, Covariate: age, sex, pre-treatment Y-BOCS, ICV, data type.

Difusivity	Tract	Mean ± SD			
		Remission (N = 19)	Non-remission (N = 14)	F value	p value
Axial	Corpus callosum – rostrum	0.00123 ± 0.00005	0.00128 ± 0.00005	6.5518	0.017
Diffusivity	Acoustic radiation – left	0.00111 ± 0.00005	0.00107 ± 0.00005	4.7257	0.039
(AD)	Cingulum bundle – dorsal – right	0.00106 ± 0.00004	0.00108 ± 0.00005	4.4786	0.044
	Superior longitudinal fasciculus II – right	0.00099 ± 0.00005	0.00101 ± 0.00004	4.2947	0.048

## 4 Discussion

This study is the first to examine both pre-CBT rsFC and white matter integrity in relation to patients’ treatment response to CBT for OCD. We identified two main findings. First, the remission group exhibited hyperconnectivity primarily between the bilateral occipital pole and lateral occipital cortex and hypoconnectivity between the occipital pole and cingulate gyrus when compared to the non-remission group. Second, although not corrected for multiple comparisons, differences in AD were observed in several tracts, including the corpus callosum rostrum, left acoustic radiation, right cingulum bundle (dorsal), and right SLF II.

### 4.1 Discussion on the results of resting-state functional connectivity

#### 4.1.1 Resting-state functional connectivity within the occipital cortex

This study explored how rsFC within the occipital cortex reflects the pathology of OCD and effects of CBT. Anatomically, the occipital pole, formed by the convergence of the superior and inferior occipital gyri, is the most posterior region of the occipital cortex and contains part of the primary visual cortex (Brodmann area 17), which is responsible for central (macular) vision (Alves et al., 2012; Rehman and Al Khalili, 2024; Hacking, last revised on 27 Jul 2022).

Functionally, the occipital cortex plays a crucial role in visual information processing, particularly in **“early visual processing.”** This includes **“retinotopy,”** which is the cortical mapping of retinal input, and **“hierarchical processing,”** in which information flows from the primary visual cortex (V1) to higher-order areas such as V2 and V3 (Genç et al., 2016). The hyperconnectivity of rsFC in the bilateral occipital cortex observed in this study likely reflects these fundamental early visual processes.

Beyond its well-established role in vision, recent research has suggested that the occipital cortex may be involved in broader neural networks relevant to OCD. For example, Geffen et al. (2022) reported a link between the occipital cortex and the DMN in OCD, extending the focus beyond the traditionally emphasized CSTC circuit. Previous studies have also reported abnormal occipital connectivity in patients with OCD. Moreira et al. (2017) found reduced connectivity between the lingual gyri and sensorimotor areas, implying disrupted visuo-somatosensory integration. Hou et al. (2014) observed decreased connectivity with the orbitofrontal cortex, suggesting impaired visual-emotional processing. Additionally, Reggente et al. (2018) demonstrated that pre-treatment connectivity within the default mode and visual networks can predict post-CBT symptom improvement.

In contrast to these findings, our study found altered FC in the occipital cortex itself, rather than between the occipital and distant brain regions. Moreover, by focusing specifically on remission following CBT, our findings emphasize the potential of occipital connectivity as a clinically meaningful neural marker of full therapeutic response.

#### 4.1.2 Resting-state functional connectivity between the posterior cingulate and occipital cortices

The posterior cingulate cortex (PCC), located in the mid-portion of the inferior parietal lobe, is a core structure within the posteromedial cortex alongside the precuneus and retrosplenial cortex (Leech and Sharp, 2014; Parvizi et al., 2006). From an anatomical perspective, the PCC receives spatial and action-related inputs from the adjacent parietal cortical areas (Rolls, 2019). Functionally, it serves as a central hub of the DMN, showing increased activation during internally focused cognitive states, including autobiographical memory retrieval, future planning, and resting conditions that involve minimal interaction with the external environment (Leech and Sharp, 2014).

Several studies have highlighted the involvement of the PCC in OCD. For example, Jang et al. (2010) demonstrated that OCD symptom dimensions are associated with distinct PCC connectivity patterns. Cleaning symptoms are associated with positive FC with the right middle frontal gyrus and negative FC with the right superior orbital gyrus, while obsession/checking symptoms showed negative FC with the bilateral middle frontal gyrus and increased connectivity with DMN-related regions, including the left middle orbital gyrus and right calcarine gyrus. Additionally, symmetry/hoarding symptoms were linked to positive FC with occipital regions, such as the cuneus and lingual gyrus. Supporting these findings, Tomiyama et al. (2023) reported that elevated fractional amplitude of low-frequency fluctuations in the PCC are associated with impaired motor response inhibition in OCD, implicating the PCC in cognitive control deficits.

Although direct rsFC between the PCC and occipital cortex has not been extensively studied, both regions have been independently implicated in OCD-related network alterations. Notably, Geffen et al. (2022) demonstrated altered FC between the visual network and DMN in patients with OCD. They revealed reduced connectivity between the lateral parietal seeds (part of the DMN) and the left inferior lateral occipital pole, as well as stronger coupling between the right lateral parietal seed and right lateral occipital cortex and precuneus. The PCC is the central hub of the DMN; therefore, our finding of reduced rsFC between the PCC and occipital cortex may be considered consistent with the results of Geffen et al. (2022), further supporting the notion of disrupted communication between the unimodal visual areas and transmodal DMN regions in OCD.

Taken together, these findings suggest that reduced PCC–occipital connectivity may reflect broader disruptions in the integration of visual information with internally directed cognitive processes. This supports the emerging view that OCD involves not only fronto-striatal dysfunction but also altered large-scale network interactions between the sensorimotor and transmodal systems.

Furthermore, these findings indicate that OCD may involve both local abnormalities in **early-stage visual processing** within the occipital cortex and **disrupted long-range connectivity** between the occipital cortex and transmodal hubs, such as the PCC. The former may reflect altered sensory encoding, whereas the latter may indicate impaired integration of visual inputs with internally directed cognitive processes, such as memory, self-referential thinking, or affective valuation. This pattern supports the notion that OCD is characterized not only by dysfunction in fronto-striatal circuits but also by abnormal interactions between **sensory and transmodal networks**, which may underlie maladaptive interpretations of visual stimuli.

#### 4.1.3 Comparison between our data and large-scale studies

This study demonstrated altered rsFC within the occipital cortex and between the PCC and occipital regions in OCD, highlighting both localized sensory abnormalities and broader network disruptions relevant to treatment response. Rather than reiterating established fronto-striatal models, our findings emphasize the role of sensory and transmodal integration in OCD, particularly in relation to CBT outcomes. The observed occipital hyperconnectivity likely reflects atypical early visual processing, whereas reduced PCC–occipital connectivity in non-remitters suggests impaired integration between visual input and internally directed cognition, a core function of the DMN. This aligns with the findings of Bruin et al. (2023), who observed altered FC between DMN regions, including the parietal cortices, and posterior brain regions encompassing the occipital cortex in OCD. Our study extends these findings by demonstrating that PCC–occipital rsFC patterns are associated with full clinical remission, suggesting their potential as markers of treatment responsiveness.

In contrast to the large-scale dysconnectivity across the DMN, frontoparietal, and salience networks reported in previous studies (Gürsel et al., 2018), our results revealed more circumscribed abnormalities within visual-transmodal interactions. This suggests that OCD may also involve deficits in integrating perceptual information with internally generated mental states. By specifically linking occipital connectivity to therapeutic outcomes, our findings suggest a promising direction for identifying neurobiological markers of remission following CBT. These results support a broader model of OCD that incorporates fronto-striatal dysfunction and altered interactions between sensory systems and higher-order networks, such as the DMN and PCC. These networks are essential for interpreting, evaluating, and regulating internally and externally derived information, particularly those relevant to OCD symptoms, such as intrusive thoughts and compulsive urges. Our findings highlight that occipital connectivity is associated with CBT remission. This suggests that the functional dynamics between the sensory and transmodal areas may serve as clinically meaningful targets for understanding and monitoring therapeutic outcomes in OCD.

### 4.2 Discussion of the tractography results

In this section, we discuss alterations in AD across four specific white matter tracts that showed group differences in this study. For each tract, we provide a brief anatomical and functional overview, review prior findings on OCD and related conditions, and consider the potential implications for CBT-related changes.

#### 4.2.1 AD value difference in the rostrum of the corpus callosum

The rostrum of the corpus callosum connects the orbital frontal cortices across hemispheres and plays a key role in interhemispheric communication (Kier and Truwit, 1997). Previous studies have reported reduced FA in this region in patients with OCD (Saito et al., 2008); however, its involvement in treatment-related plasticity remains underexplored.

#### 4.2.2 AD value difference in acoustic radiation

Acoustic radiation carries auditory signals from the thalamus to the auditory cortex and is critical for sensory processing (Maffei et al., 2019). A key component of the auditory system, it forms the thalamocortical pathway that transports auditory signals from the medial geniculate nucleus of the thalamus to the transverse temporal gyrus in the superior temporal lobe (Maffei et al., 2019). Increased RD differences have been observed in left acoustic radiation in children with tic disorders (Kang et al., 2024); however, its relationship to OCD remains unclear and warrants further investigation.

#### 4.2.3 AD value difference in the cingulum bundle – dorsal

The dorsal cingulum connects the medial prefrontal and parietal areas and is involved in cognitive control and emotion regulation (Bubb et al., 2018). In pediatric patients with OCD, AD was found to be significantly higher in the left dorsal cingulum bundle, and greater FA in this area is associated with better performance on response inhibition and cognitive control tasks, such as the Stroop test and trail-making test (Gruner et al., 2012), suggesting that this tract may be sensitive to CBT-related changes in executive function.

#### 4.2.4 AD value difference in the superior longitudinal fasciculus II

The SLF II is a subcomponent of the SLF that connects the angular gyrus in the inferior parietal lobule with the caudal–lateral prefrontal cortex. It plays a key role in visual attention and spatial orientation by integrating perceptual input with executive control (Janelle et al., 2022). Given these functions, the SLF II is of particular interest in OCD research, in which impairments in visuospatial cognition are frequently reported. For example, patients with OCD have shown reduced performance on spatial organization and navigation tasks, such as Money’s Road Map Test and Block Design (Boone et al., 1991; Zielinski et al., 1991), as well as difficulties in visuospatial construction and memory on the Rey–Osterrieth Complex Figure Test (Deckersbach et al., 2000; Savage et al., 2000). Moreover, deficits have been observed in spatial transformation, such as mental rotation, indicating possible dysfunction at the perceptual–transformational level (Moritz et al., 2001). These cognitive impairments may reflect underlying white matter abnormalities. Diffusion studies have identified increased AD in the bilateral SLF in pediatric OCD (Jayarajan et al., 2012), and associations between FA in the left SLF and visuospatial and working memory abilities have been previously reported (Koshiyama et al., 2020). Furthermore, increased RD in the right SLF has been observed in adult patients with OCD (Magioncalda et al., 2016). Taken together, findings from cognitive assessments and diffusion MRI studies suggest that impairments in visuospatial processing, accompanied by alterations in higher-order cognitive functions in OCD, may stem from structural abnormalities in neural networks. In particular, the SLF II appears to play a critical role as part of the network involved in retaining, transforming, and manipulating visuospatial information.

#### 4.2.5 Comparison between our data and large-scale studies

The ENIGMA-OCD Working Group previously demonstrated significant reductions in FA in adult patients with OCD, particularly in the sagittal stratum and posterior thalamic radiation (Piras et al., 2021). Furthermore, diffusion white matter features contributing to OCD classification, when compared to healthy controls, include anisotropy and diffusivity estimates of white matter in the internal capsule, thalamic radiation, and uncinate fasciculus (B.-G. Kim et al., 2024). In contrast, the present study focused on AD values extracted from the corpus callosum rostrum, left acoustic radiation, right cingulum bundle (dorsal), and right SLF II, which have not been highlighted in large-scale studies conducted by the ENIGMA-OCD Working Group.

This divergence may stem not only from methodological differences but also from distinct research aims. While the ENIGMA-OCD studies focused on classifying patients with OCD relative to healthy controls, our study examined treatment responsiveness within the OCD population to specifically investigate remission following CBT.

These differences in research focus were paralleled by differences in analytical methodology. The ENIGMA-OCD study used TBSS, whereas our study used TRACULA. TBSS is a voxel-based analysis method that identifies group-level differences across the entire white matter skeleton, whereas TRACULA is a tract-based approach that reconstructs predefined white matter pathways using anatomical priors. This targeted analysis enables a more functionally specific interpretation of structural abnormalities, potentially leading to a more nuanced understanding of the pathology associated with OCD and its response to CBT. This methodological focus could offer insights into treatment mechanisms that may not be captured as readily in larger, more generalized studies.

### 4.3 Multimodal evaluation of connectivity related to CBT outcomes

#### 4.3.1 Studies focusing on CBT in relation to rsFC and white matter integrity

The findings of this study related to CBT can be compared to previous research on the effects of CBT on rsFC and white matter integrity. For example, pretreatment multivariate connectivity in the DMN and the visual network was found to significantly predict OCD symptom reduction after four weeks of intensive CBT (Reggente et al., 2018). Furthermore, decreased rsFC between the basolateral amygdala and ventromedial prefrontal cortex (vmPFC) was found to predict better CBT outcomes in patients with OCD (Fullana et al., 2017). Another previous study (J. Gao et al., 2021) revealed that CBT leads to decreased rsFC between the amygdala subregions and visual association cortices and increased rsFC between the amygdala and right inferior parietal lobe. The involvement of the visual cortex reported in this study aligns with our findings, particularly in terms of the observed hyperconnectivity within the occipital cortex described in Section 4.1.1. This provides evidence that early visual processing areas may play a role in treatment-related changes in OCD and highlights occipital rsFC as a potential neural marker of remission following CBT. Although previous studies have implicated amygdala connectivity in CBT outcomes, we found no significant association with the amygdala (see Section 4.2.7), suggesting that emotional processing may play a less central role in the mechanisms captured by our analysis.

Furthermore, Zhong et al. (2019 reported that following CBT, patients with OCD exhibited increased FA values in the right middle frontal gyrus, left orbitofrontal cortex, right cerebellum, and left middle temporal gyrus, whereas FA decreased in the right putamen compared to pretreatment levels. However, Brecke et al. (2021) found no significant association between baseline FA in ROIs and changes in Y-BOCS scores three months post-CBT, suggesting challenges in detecting clear associations with clinical improvements.

These mixed results indicate that detecting significant changes in white matter integrity and rsFC in relation to CBT can be complex. While this study focused on pretreatment tractography to evaluate treatment response, future research could benefit from incorporating functional interpretations along with structural imaging to provide a more comprehensive understanding of the neurobiological mechanisms underlying the impact of CBT on OCD. In the following section, we explore this integrated perspective by combining functional and structural connectivity analyses to better characterize treatment-related brain changes.

#### 4.3.2 Evaluating functional and structural connectivity derived from multimodality

Building on previous studies that examined functional or structural changes associated with CBT separately, the current study aimed to bridge these perspectives using a multimodal approach to evaluate both rsFC and white matter connectivity. The next step is to integrate findings from the perspectives of both functional and structural connectivity to identify common patterns. The rsFC results suggest a link to visual information processing, while the tractography results highlight the involvement of the SLF-II in visuo-perceptual processing. Together, these functional and structural connectivity findings broadly suggest the possible role of visual processing in OCD remission following CBT.

While the main findings have emphasized the role of visual processing, previous research has also considered emotional influences on visual perception. Reggente et al. (2018) examined how emotionally charged stimuli can trigger visual information processing, and excessive vigilance might enhance obsessive attention to seemingly unimportant environmental stimuli (e.g., a dirty doorknob) or irrelevant details (Yovel et al., 2005). They investigated whether rsFC in and around the amygdala could predict treatment outcomes; however, their data did not lead to significant improvements in predictive performance. Given that previous studies have highlighted the role of emotional processing, and amygdala activity in particular, in modulating visual perception (e.g., Reggente et al., 2018), we hypothesized that amygdala connectivity might differ between remission and non-remission groups. Based on a similar hypothesis, we conducted a seed-to-voxel analysis using the amygdala as the seed. However, we found no significant differences in rsFC between the remission and non-remission groups. This lack of significant findings may reflect the functional heterogeneity of the amygdala, suggesting that examining its specific subregions may yield more precise insights into its role in OCD. However, given the limited influence of emotional factors on visual processing observed in our study, the amygdala may play a more peripheral role in CBT-related neural changes.

Taken together, these findings highlight the value of a multimodal approach in uncovering the neurobiological mechanisms underlying treatment response to CBT in OCD, particularly those involving visual processing pathways.

### 4.4 Image analysis methods

#### 4.4.1 fc-MVPA

Several previous studies exploring rsFC have utilized either ROI-to-ROI analysis (Calzà et al., 2019; Kashyap et al., 2021; Takagi et al., 2017), which evaluates FC between predefined ROIs, or seed-to-voxel analysis (Geffen et al., 2022; Kwon et al., 2024), which assesses whole-brain FC using seeds arbitrarily selected from prior studies. Although he analysis may appear to cover the whole brain, ROI-to-ROI analysis based on standard atlases or ROIs derived from previous studies is hypothesis-driven and susceptible to selection bias when choosing ROIs (Kurita et al., 2023). Previous rsFC studies have commonly involved arbitrary selection of ROIs or seed areas based on prior literature or hypotheses, regardless of whether they employed ROI-to-ROI (Calzà et al., 2019; Kashyap et al., 2021; Takagi et al., 2017) or seed-to-voxel analysis (Geffen et al., 2022; Kwon et al., 2024). Despite differences in analytical scope, this reliance on predefined or literature-derived regions makes both approaches prone to selection bias (Kurita et al., 2023). In contrast, fc-MVPA is data-driven, which reduces selection bias in choosing ROIs. This method allows for a more comprehensive understanding and unbiased exploration of brain connectivity, as it is not confined to a specific set of ROIs (Mateu-Estivill et al., 2024). Moreover, fc-MVPA potentially enables the detection of rsFC beyond the well-known CSTC circuit (Stein et al., 2019).

In our study, we also conducted ROI-to-ROI analysis but did not identify any significant rsFC (connection threshold: *p* < *0.001* uncorrected for multiple comparisons; cluster threshold: *p* < *0.001* cluster-level FDR corrected for multiple comparisons). These results further support the notion that fc-MVPA can uncover rsFC that may otherwise remain undetected through traditional analysis methods.

#### 4.4.2 Quality control and reinitialization in TRACULA

We adhered to the Quality Control Pipeline for Probabilistic Reconstruction of White-Matter Pathways (X. He et al., 2021) to enhance the detection and recovery of incomplete white matter tracts. This pipeline includes the following steps:

(1) Performing a visual inspection of eddy current-corrected diffusion-weighted images,
(2) Conducting an automated evaluation of color-encoded FA images,
(3) Assessing the volume of each tract saved in the TRACULA output file, and
(4) Reprocessing tracts with volumes smaller than a specified threshold.

For tracts that remain partially reconstructed, Steps 5 and 6 in the original pipeline involve minimal manual editing of control points and reinitiating TRACULA, respectively. However, we excluded Step 5 (i.e., manual control point editing) to maintain a fully automated processing pipeline and only applied Step 6, reinitialization of TRACULA. Although a few tracts required reinitialization, only 1.4 % of the total (20 out of 1386; 42 tracts × 33 participants) were ultimately flagged as poorly reconstructed. Given this small proportion, we determined that their impact on the overall results would be minimal. Additionally, considering the potential for variability and bias introduced by manual editing of control points (Step 5), we chose to skip this step in favor of maintaining consistency and reproducibility across participants.

## 5 Conclusion

In conclusion, to our knowledge, this is the first study to evaluate both functional and structural connectivity before treatment in relation to CBT outcomes, specifically remission, following CBT for OCD. In comparisons between the remission and non-remission groups, significant differences were observed in FC, particularly involving the occipital cortex. We also found differences in white matter integrity in the corpus callosum rostrum, left acoustic radiation, right cingulum bundle (dorsal), and right SLF II. Furthermore, our findings suggest that alterations in visual processing networks, which were consistently observed across both functional and structural modalities, may serve as potential markers of CBT responsiveness in OCD.

## 6 Limitations

This study had some limitations. First, the sample size was relatively small, with only 33 patients with OCD participating. Based on our findings, we posit that visual processing may influence remission after CBT for OCD; however, larger cohort studies are needed to validate this theory. Second, our analysis did not account for or exclude the medications that the patients were taking, leaving the potential effects of these medications on brain function and white matter unknown. This will require further investigation. In addition to participant-related factors, data acquisition inconsistencies warrant consideration. Third, we used two types of rsfMRI and DTI data obtained from the same machine under different conditions. Although no significant differences were observed between these two data types, and they were included as covariates, future studies should prioritize using the same data type for analysis to ensure consistency. Finally, we did not perform the manual editing step of the Quality Control Pipeline (X. He et al., 2021). If semi-automatic recovery through manual editing were applied to improve the quality of the reconstructed white matter tracts, we would likely obtain more accurate assessments of white matter integrity. Despite these limitations, the findings of this study offer important insights into the neural mechanisms underlying treatment response in OCD and lay the groundwork for future multimodal imaging research.

## CRediT authorship contribution statement

**Yuki Ikemizu:** Writing – review & editing, Writing – original draft, Visualization, Validation, Software, Resources, Methodology, Investigation, Funding acquisition, Formal analysis, Data curation, Conceptualization. **Yuko Isobe:** Writing – review & editing, Methodology, Investigation. **Yusuke Sudo:** Writing – review & editing, Methodology. **Junko Ota:** Methodology. **Ritu Bhusal Chhatkuli:** Methodology. **Tubasa Sasaki:** Writing – review & editing, Methodology, Investigation, Data curation. **Kohei Kurita:** Writing – review & editing, Methodology, Investigation, Data curation. **Tokiko Yoshida:** Investigation, Data curation. **Koji Matsumoto:** Resources, Methodology, Investigation, Data curation. **Masaru Kuno:** Writing – review & editing, Resources. **Naoko Kato:** Writing – review & editing, Resources. **Akiko Nakagawa:** Writing – review & editing, Resources. **Eiji Shimizu:** Writing – review & editing, Supervision, Investigation, Funding acquisition. **Yoshiyuki Hirano:** Writing – review & editing, Visualization, Supervision, Project administration, Methodology, Investigation, Funding acquisition, Formal analysis, Data curation, Conceptualization.

## Declaration of generative AI and AI-assisted technologies in the writing process

During the preparation of this work, the author used ChatGPT-4o for English proofreading. After utilizing this tool, the author reviewed and edited the content as needed and takes full responsibility for the final content of the publication.

## Role of the funding source

This work was supported by the 10.13039/100009619AMED Brain/MINDS Beyond Program [grant number JP18dm0307002] and the 10.13039/501100001691JSPS KAKENHI Grants [grant numbers 19K03309, 22H01090, 23K22361, 23K07004, and 24K21493].

## Declaration of competing interest

The authors declare no conflicts of interest associated with this manuscript.

## Data Availability

Data will be made available on request.
