# Supplementary material for: Pre-CBT resting-state connectivity and white matter integrity in OCD remission: A multimodal MRI study
Source: Neuroimage Rep. 2025 Jul 8;5(3):100275. doi: 10.1016/j.ynirp.2025.100275 (PMC12489777; doi:10.1016/j.ynirp.2025.100275)
Supplement: Multimedia component 1 [file mmc1.docx]

Supplementary Method

1. Multivariate pattern analysis (MVPA)

At the first level, functional connectivity (FC) was calculated for each voxel within the gray matter template, generating an M (subjects) × N (brain voxels) matrix per seed voxel. Dimensionality reduction was then performed using principal component analysis (PCA) to maximize between-subject differences while reducing the spatial components (Whitfield-Gabrieli et al., 2016). The five strongest component score sets were retained, forming an M × C (components) matrix, ensuring a conservative subject-to-component ratio of 5:1 to 10:1. At the second level, an omnibus F-test was conducted on the three components for each voxel to identify multivariate voxel patterns that distinguish between the remission and non-remission groups (Weber-Goericke & Muehlhan, 2023).

References

Weber-Goericke, F., & Muehlhan, M., 2023. High and low worriers do not differ in unstimulated resting-state brain connectivity. *Scientific Reports*, *13*(1), 3052. https://doi.org/10.1038/s41598-023-28333-5
